# Supplementary material for: Inhibitors of the Oncogenic PA2G4-MYCN Protein-Protein Interface
Source: Cancers (Basel). 2023 Mar 17;15(6):1822. doi: 10.3390/cancers15061822 (PMC10046377; doi:10.3390/cancers15061822)
Supplement: Supplementary file 1 [file cancers-15-01822-s001.zip › cancers-2238268-supplementary.pdf]

A

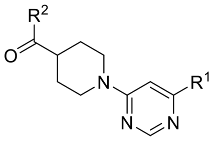

Compound ID Gen 1	R¹	R²	BE(2)C (µM)	Kelly (µM)	MRC5 (µM)	WI36 (µM)	Therapeutic window
SVI-3556	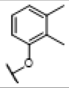	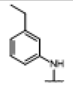	100.00	35.00	100.00	100.00	1.48
SVI-3557	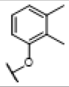	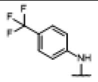	26.00	30.00	100.00	80.00	3.21
SVI-3558	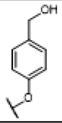	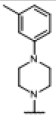	50.00	100.00	100.00	100.00	1.3
SVI-3559	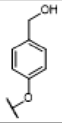	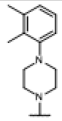	100.00	100.00	100.00	100.00	1
SVI-3560	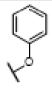	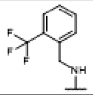	100.00	100.00	100.00	100.00	1
SVI-3561	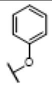	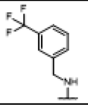	100.00	100.00	100.00	100.00	1
SVI-3562	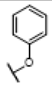	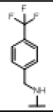	100.00	100.00	100.00	100.00	1
SVI-3563	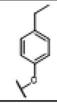	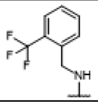	100.00	100.00	100.00	100.00	1
SVI-3564	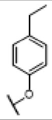	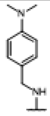	100.00	100.00	100.00	100.00	1
SVI-3565	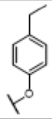	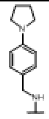	100.00	100.00	100.00	100.00	1
SVI-3566	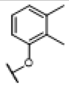	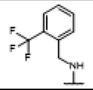	100.00	100.00	100.00	100.00	1
SVI-3568	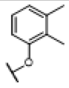	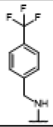	28.84	32.77	38.65	48.02	1.4
SVI-3569	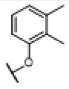	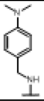	50.00	75.00	100.00	100.00	1.6

B

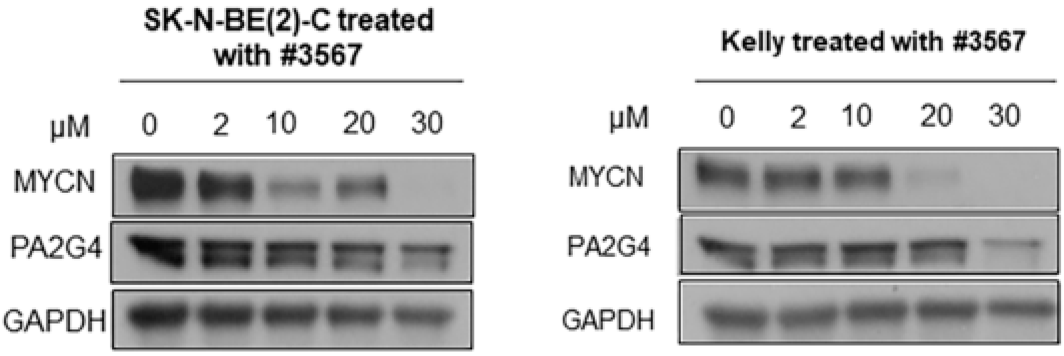
SK-N-BE(2)-C treated with #3567
μM 0 2 10 20 30
MYCN
PA2G4
GAPDH
Kelly treated with #3567
μM 0 2 10 20 30
MYCN
PA2G4
GAPDH

C

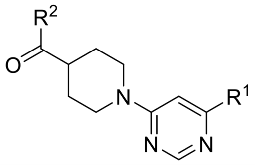

Compound ID Gen 2	R¹	R²	BE(2)C (µM)	Kelly (µM)	MRC5 (µM)	WI36 (µM)	Therapeutic window
SVI-5307	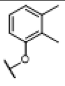	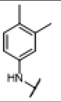	100.00	100.00	100.00	100.00	1
SVI-5308	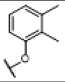	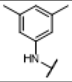	40.00	37.00	100.00	100.00	2.59
SVI-5309	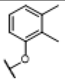	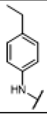	100.00	100.00	100.00	100.00	1
SVI-5310	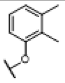	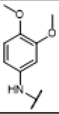	100.00	100.00	100.00	100.00	1
SVI-5311	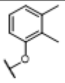	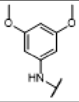	40.00	32.00	100.00	100.00	2.7
SVI-5312	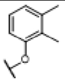	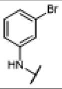	25.00	28.00	100.00	100.00	3.77
SVI-5313	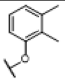	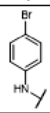	32.00	39.00	100.00	100.00	1.9
SVI-5314	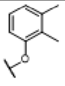	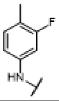	35.00	80.00	100.00	100.00	1.734
SVI-5315	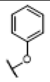	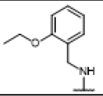	100.00	100.00	100.00	100.00	1
SVI-5316	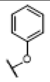	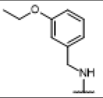	60.00	60.00	75.00	75.00	1.25
SVI-5317	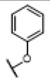	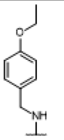	55.00	30.00	45.00	58.00	1.21
SVI-5318	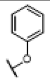	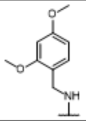	100.00	70.00	100.00	100.00	1.17
SVI-5319	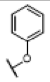	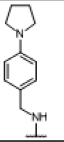	100.00	100.00	100.00	100.00	1

SVI-5320	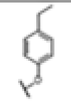	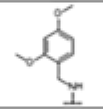	100.00	100.00	100.00	100.00	1
SVI-5321	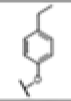	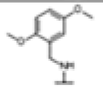	70.00	60.00	100.00	100.00	1.54
SVI-5322	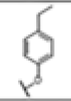	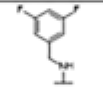	100.00	100.00	100.00	100.00	1
SVI-5323	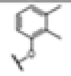	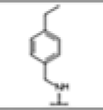	4.00	7.00	10.00	30.00	2.2
SVI-5324	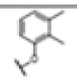	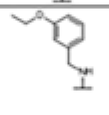	32.00	52.00	100.00	100.00	2.4
SVI-5325	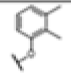	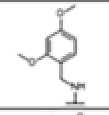	65.00	60.00	100.00	100.00	1.6
SVI-5326	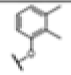	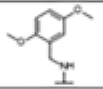	53.00	40.00	100.00	100.00	2.2
SVI-5327	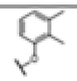	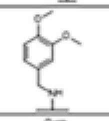	100.00	100.00	100.00	100.00	1
SVI-5328	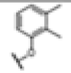	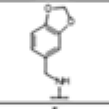	100.00	70.00	100.00	100.00	1.176
SVI-5329	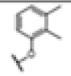	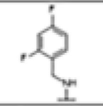	25.00	12.00	22.00	58.00	2.16
SVI-5330	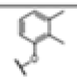	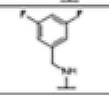	55.00	65.00	100.00	100.00	1.67
SVI-5331	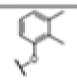	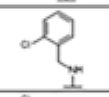	55.00	30.00	75.00	100.00	2.1
SVI-5332	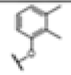	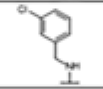	30.00	35.00	100.00	100.00	3.07

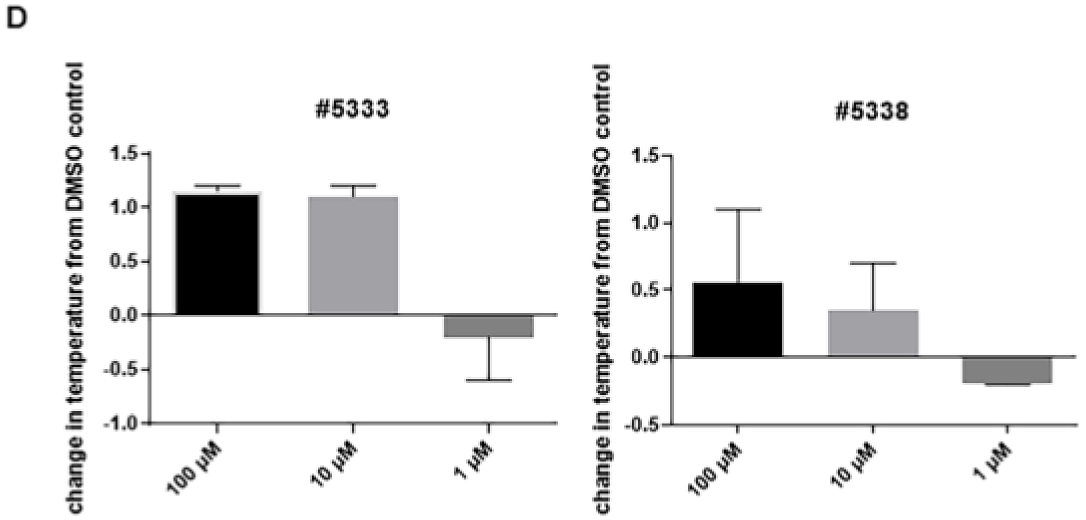

Figure S1. (**A**) Structure to activity relationships (SAR) of WS6 analogues by comparisons of IC50 concentrations for treated neuroblastoma cells (SK-N-BE(2)-C and Kelly cells) and normal fibroblasts (MRC5 and WI36 cells) for R1 and R2 substituents of WS6. (**B**) Western blot showing a concentration-dependent decrease in MYCN and PA2G4 protein expression following #3567 treatment of SK-N-BE(2)-C and Kelly neuroblastoma cells. (**C**) SAR of #3567 analogues with R1 and R2 substituents by comparisons of IC50 concentrations for treated neuroblastoma and normal fibroblast cells. (**D**) Differential Scanning fluorimetry (DSF) of #5333 and #5338 changing the melting temperature of the PA2G4 protein, relative to DMSO control.

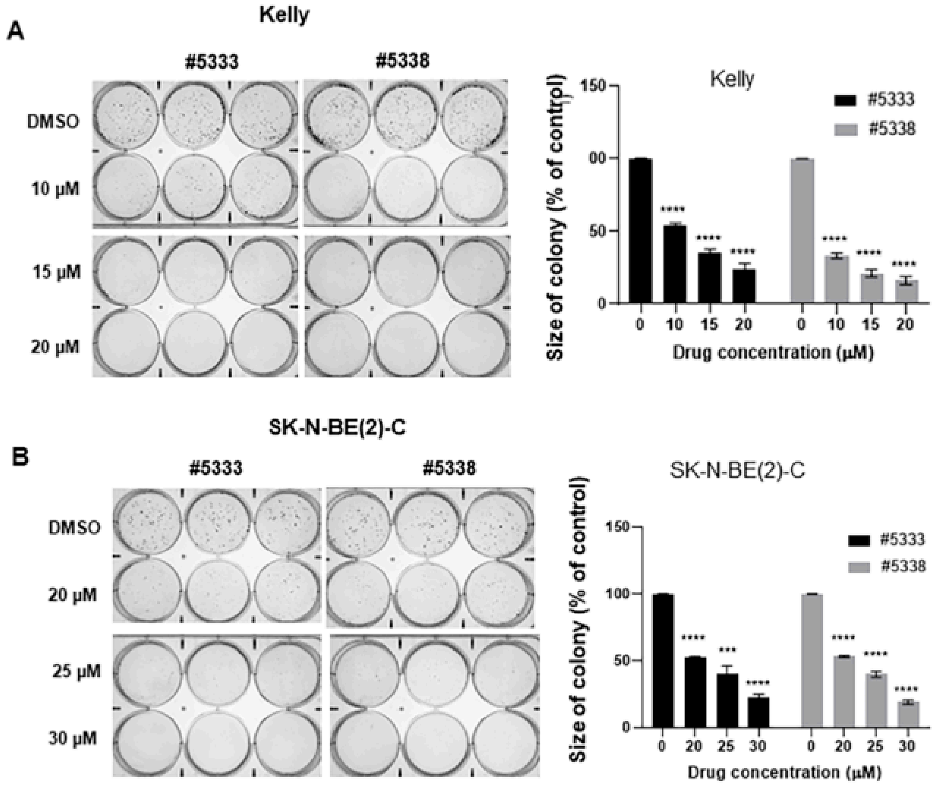

Figure S2. Representative colony formation plate images and the size of the colonies of (**A**) Kelly and (**B**) SK-N-BE(2)-C neuroblastoma cells treated with #5333 or #5338 for 2 weeks at a range of concentrations. "***" is p value < 0.001 and "****" is p value <0.0001 when compared with no treatment control.

A

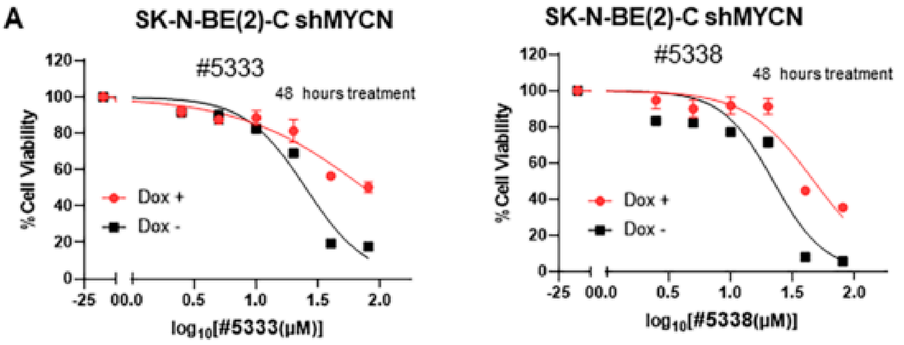

SK-N-BE(2)-C shMYCN

B

Dox (2μg/ml)	-	+	p value
MYCN	+	-	
#5333 IC50 (μM)	25.04	74.34	< 0.0001
#5338 IC50 (μM)	22.04	46.81	< 0.0001

C

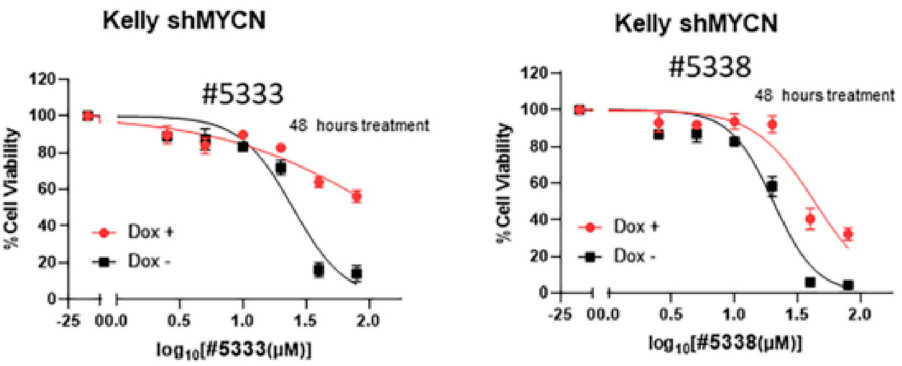

D

Kelly shMYCN

Dox (2μg/ml)	-	+	p value
MYCN	+	-	
#5333 IC50 (μM)	24.84	117.6	< 0.0001
#5338 IC50 (μM)	20.31	43.15	< 0.0001

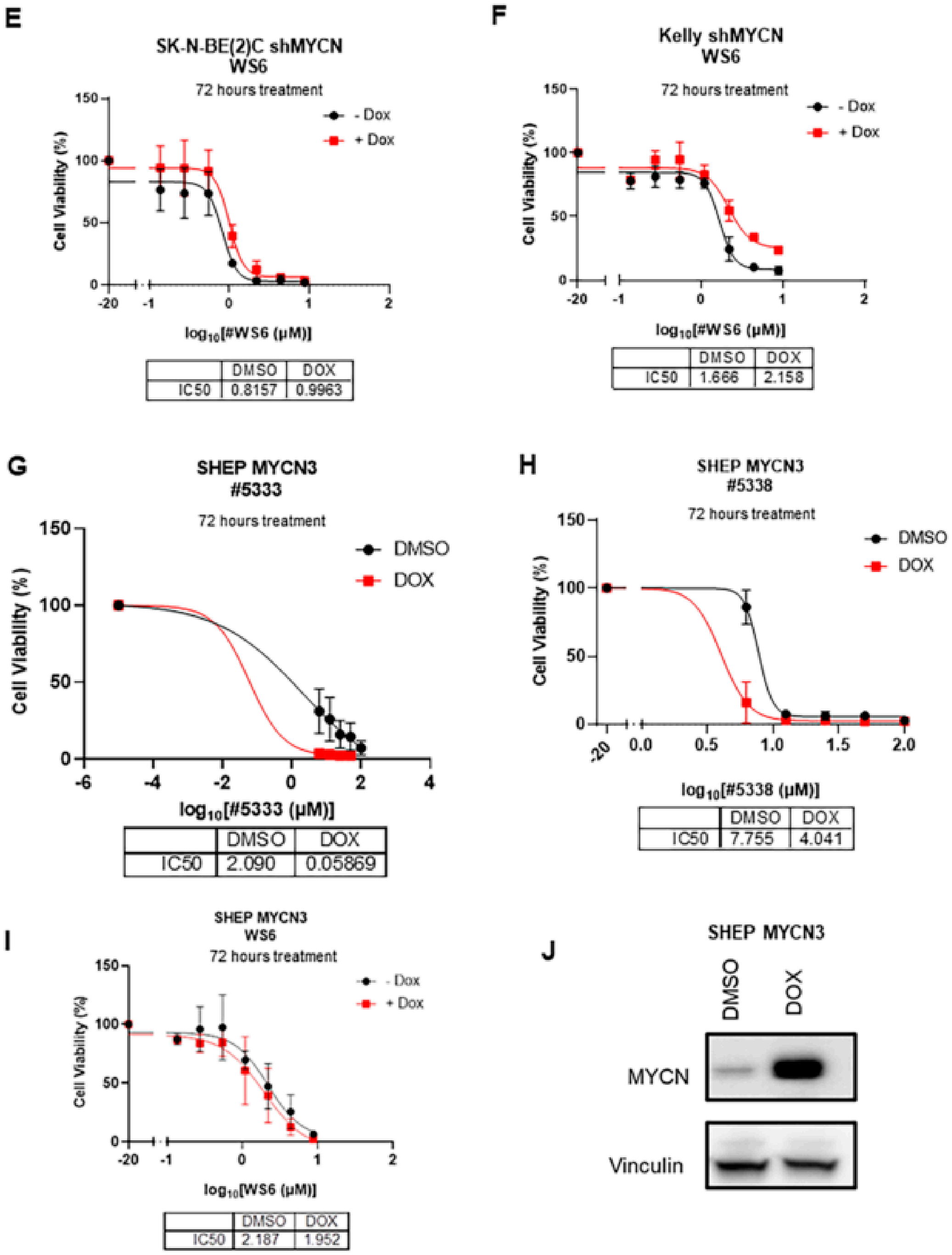

Figure S3. (**A**) Alamar blue assays comparing cell viability of SK-N-BE(2)-C shMYCN cells with Dox-inducible inhibition of MYCN expression using stable shMYCN, followed by 48 hours either #5333 or #5338 treatment. (**B**) IC50 comparisons, with and without Dox, of SK-N-BE(2)-C shMYCN treated with either #5333 or #5338 for 48 hours. (**C**) Alamar blue assays comparing cell viability of Kelly shMYCN cells with Dox-inducible inhibition of MYCN expression using stable shMYCN, followed by 48 hours of either #5333 and or #5338 treatment. (**D**) IC50 of comparisons, with and without Dox, of #5333 and #5338 treated SK-N-BE(2)-C shMYCN cells for 48 hours. (**E**,**F**) Alamar blue assays comparing cell viability of BE(2)-C shMYCN and Kelly shMYCN cells with Dox-inducible inhibition of MYCN expression, followed by 72 hours of WS6 treatment. (**G–I**) IC50 of comparisons, with and without Dox, of #5333, #5338 or WS6 treated SHEP MYCN3 cells for 72 hours. (**J**) Western blot measuring MYCN expression in Dox-inducible SHEP MYCN3 cells treated with DMSO or 2 μg/ml of Dox for 96 hours.

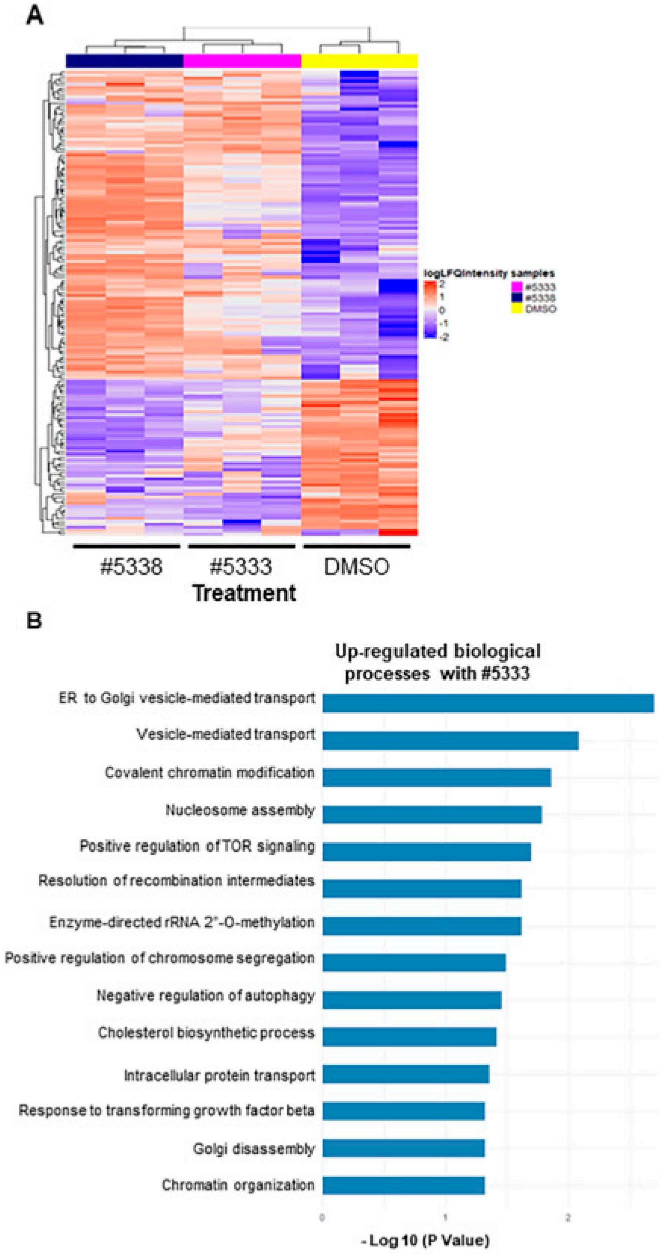
A
logLFQintensity samples
2
1
0
-1
-2
#5333
#5338
DMSO
#5338
#5333
DMSO
Treatment
B
Up-regulated biological processes with #5333
ER to Golgi vesicle-mediated transport
Vesicle-mediated transport
Covalent chromatin modification
Nucleosome assembly
Positive regulation of TOR signaling
Resolution of recombination intermediates
Enzyme-directed rRNA 2'-O-methylation
Positive regulation of chromosome segregation
Negative regulation of autophagy
Cholesterol biosynthetic process
Intracellular protein transport
Response to transforming growth factor beta
Golgi disassembly
Chromatin organization
0
1
2
- Log 10 (P Value)

C

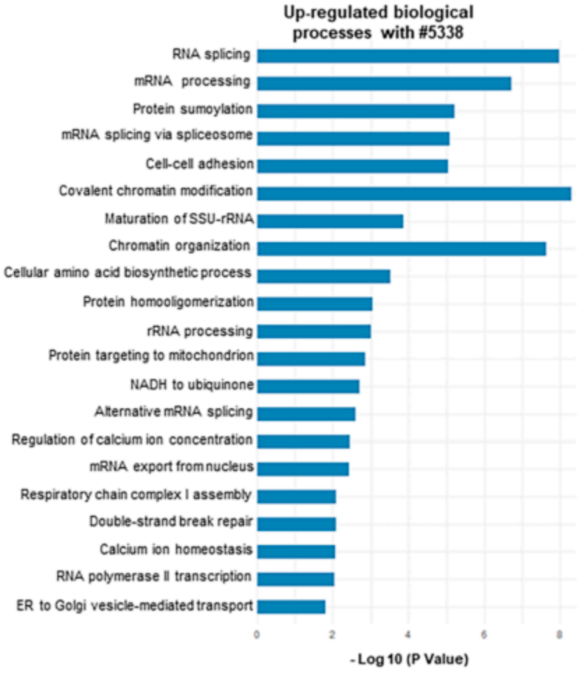
Up-regulated biological processes with #5338
RNA splicing
mRNA processing
Protein sumoylation
mRNA splicing via spliceosome
Cell-cell adhesion
Covalent chromatin modification
Maturation of SSU-rRNA
Chromatin organization
Cellular amino acid biosynthetic process
Protein homooligomerization
rRNA processing
Protein targeting to mitochondrion
NADH to ubiquinone
Alternative mRNA splicing
Regulation of calcium ion concentration
mRNA export from nucleus
Respiratory chain complex I assembly
Double-strand break repair
Calcium ion homeostasis
RNA polymerase II transcription
ER to Golgi vesicle-mediated transport
0
2
4
6
8
- Log 10 (P Value)

D

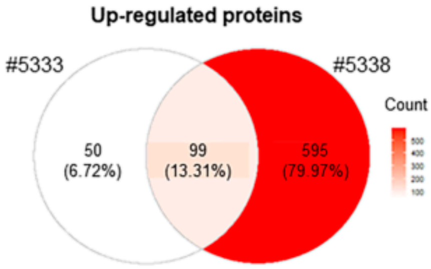
Up-regulated proteins
#5333
#5338
Count
50
(6.72%)
99
(13.31%)
595
(79.97%)

E

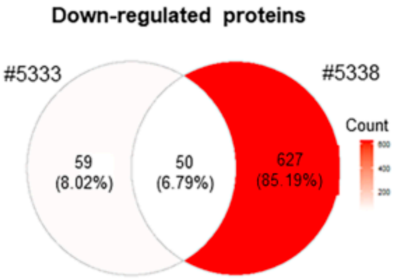
Down-regulated proteins
#5333
#5338
Count
59
(8.02%)
50
(6.79%)
627
(85.19%)

F

	Name	Description	Fold change		P values	
			#5333	#5338	#5333	#5338
Down-regulated	CLNS1A	Chloride Nucleotide-Sensitive Channel 1A	-2.08493	-3.944930818	0.000247219	3.13E-06
	CKS2	CDC28 Protein Kinase Regulatory Subunit 2	-2.17347	-3.386981249	0.000905252	4.75E-05
	GATA2	GATA Binding Protein 2	-4.19887	-5.540437872	0.005438024	0.00204185
	TTC4	Tetratricopeptide Repeat Domain 4	-2.69447	-4.626752736	0.043778462	0.006359681
	POLR3A	RNA Polymerase III Subunit A	-1.65405	-1.893427262	0.025340276	0.008550249
	IGBP1	Immunoglobulin Binding Protein 1	-2.67586	-2.281527432	0.016150433	0.034372337
	MLLT11	MLLT11, Transcription Factor 7 Cofactor	-2.20381	-5.815890069	0.009560792	7.84E-05
Up-regulated	BCAP29	B Cell Receptor Associated Protein 29	1.505247	2.751083636	0.000166691	2.67E-07
	ASNS	Asparagine Synthetase (Glutamine-Hydrolyzing)	1.860899	2.329467173	5.31E-06	5.64E-07
	GOLGA5	Golgin A5	1.55617	2.099433367	0.001599758	5.86E-05
	BAZ1B	NA	3.116658	6.062866266	0.005504928	0.000367455
	CYP51A1	Cytochrome P450 Family 51 Subfamily A Member 1	1.917199	1.504203751	0.000172959	0.003031716
	PTTG1IP	PTTG1 Interacting Protein	6.364292	24.5900029	0.045955544	0.003760453
	STC2	Stanniocalcin 2	1.872544	1.666706414	0.001482229	0.004710286
	HMGCR	NA	2.281527	3.340351678	0.035199837	0.006121218
	DNAJB12	NA	2.675855	2.514026749	0.021226897	0.02754231
	NUDT2	Nudix Hydrolase 2	5.578975	4.823231311	0.022201847	0.03177869

Figure S4. (**A**) Heat map of the differentially expressed proteins in SK-N-BE(2)-C cells treated with 2 x the IC50 concentrations of #5333, #5338, and incubated for 72 hours, compared to DMSO. (**B**) and (**C**) Up-regulated GO biological processes -log10 p value bar plots for #5333 and #5338. (**D**) and (**E**) Venn diagrams of up-regulated and down-regulated proteins following treatment of SK-N-BE(2)-C cells by #5333 and #5338 for 72 hours, showing proportionate overlap. (**F**) Table showing proteins with the highest fold changes in expression following the treatment of SK-N-BE(2)-C cells with #5333 and #5338 which were also MYC target genes.

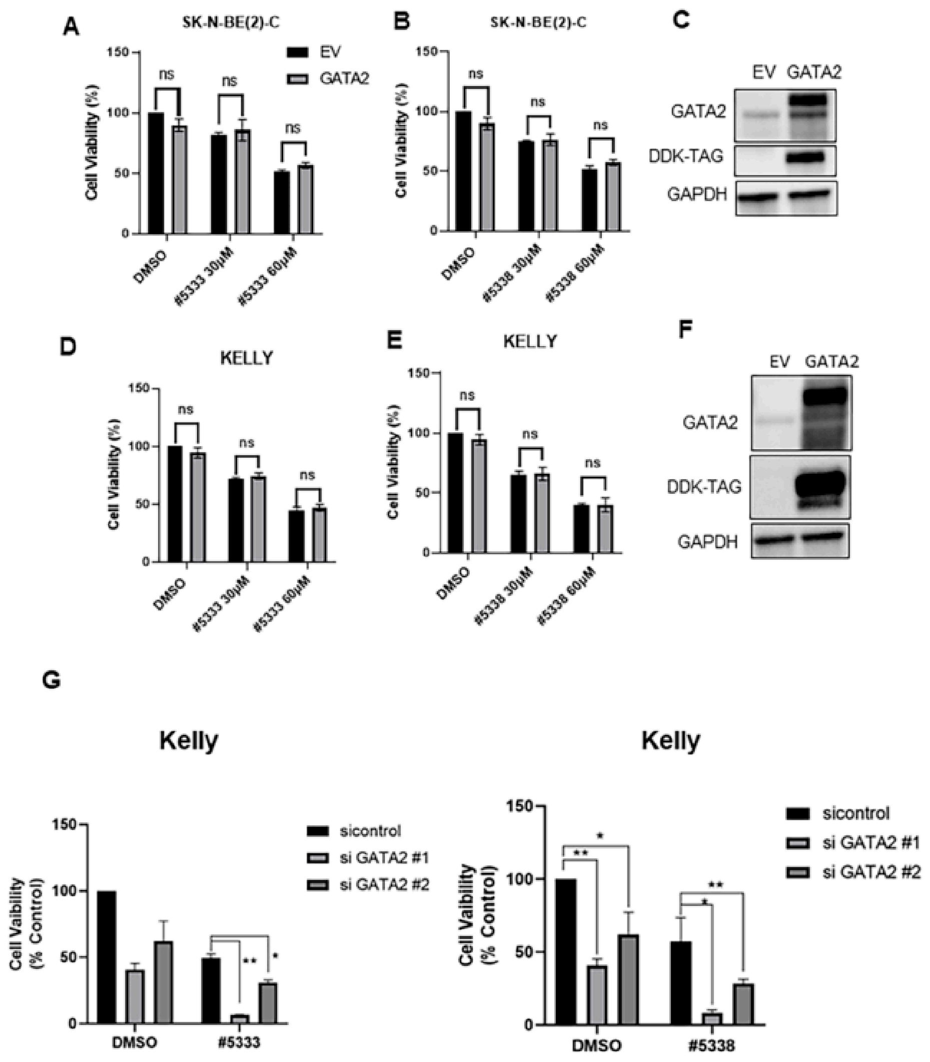

Figure S5. (**A**) and (**B**) Cell viability was measured by the Alamar blue assay in SK-N-BE(2)-C cells following overexpression of GATA2 plasmid DNA for 72 hours and treatment with #5333 or #5338 for 48 hours. (**C**) Confirmation of overexpression of GATA2 in SK-N-BE(2)-C cells detected by DDK-tag and GATA2 antibodies. (**D**) and (**E**) Cell viability was measured by the Alamar blue assay in Kelly cells following overexpression of GATA2 plasmid DNA for 72 hours and treatment with #5333 and #5338 for 48 hours. (**F**) Confirmation of overexpression of GATA2 in Kelly cells detected by DDK-tag and GATA2 antibodies. (**G**) Kelly cell viability was measured after transfected with siRNA control and two GATA2 siRNAs (siRNA#1 and #2) for 96 hours, and treated with #5333 (26.5 μM) or #5338 (23.3 μM) for 72 hours using Alamar Blue assay. "*" is p value < 0.05, "**" is p value < 0.01 when compared to DMSO treated control, ns: non-significant.
