# Supplementary figures and images for: Inhibitors of the Oncogenic PA2G4-MYCN Protein-Protein Interface
Source: Cancers (Basel). 2023 Mar 17;15(6):1822. doi: 10.3390/cancers15061822 (PMC10046377; doi:10.3390/cancers15061822)

Figure 2E

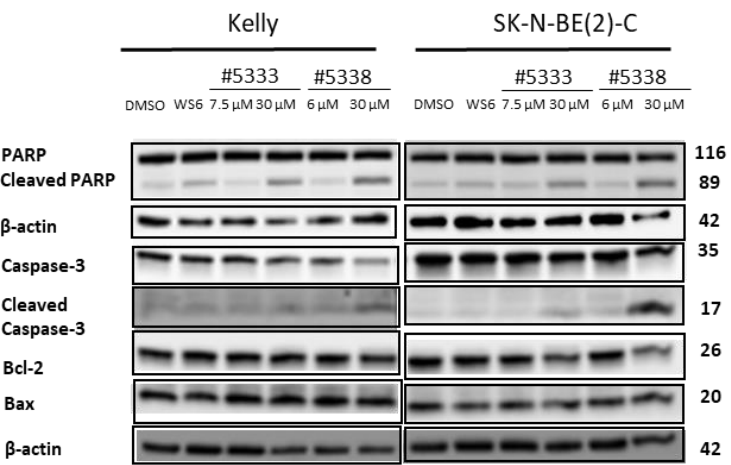

Repeat 1

Repeat 2

Repeat 3

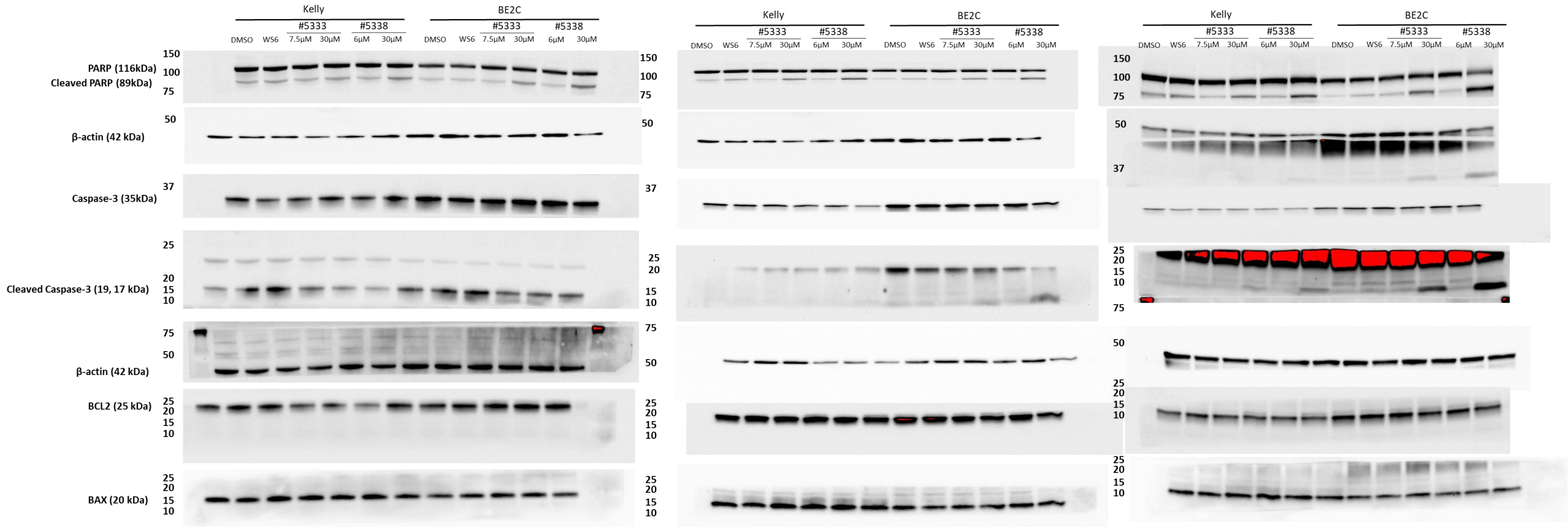

Figure 3 A

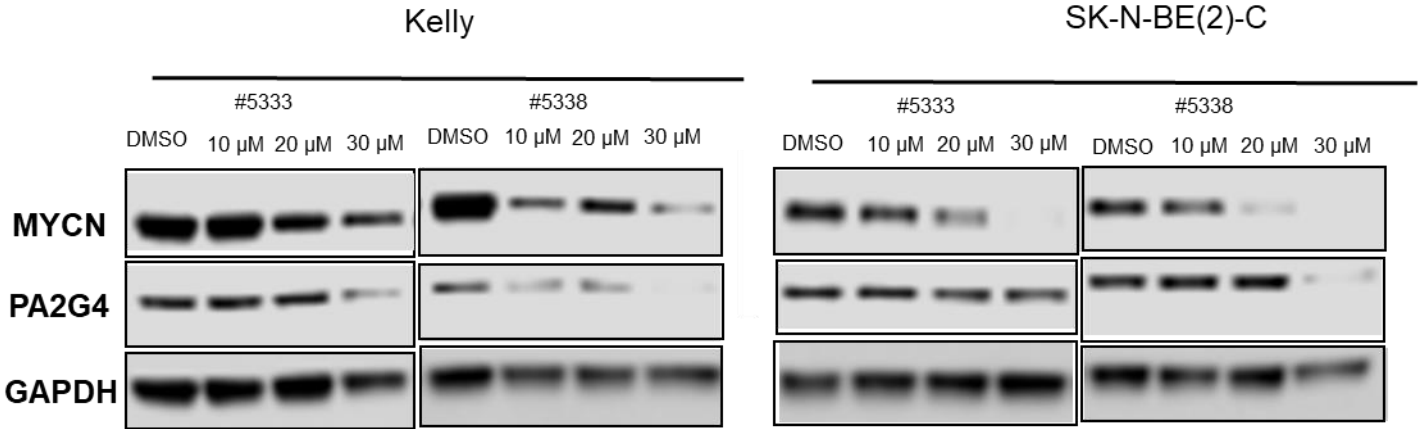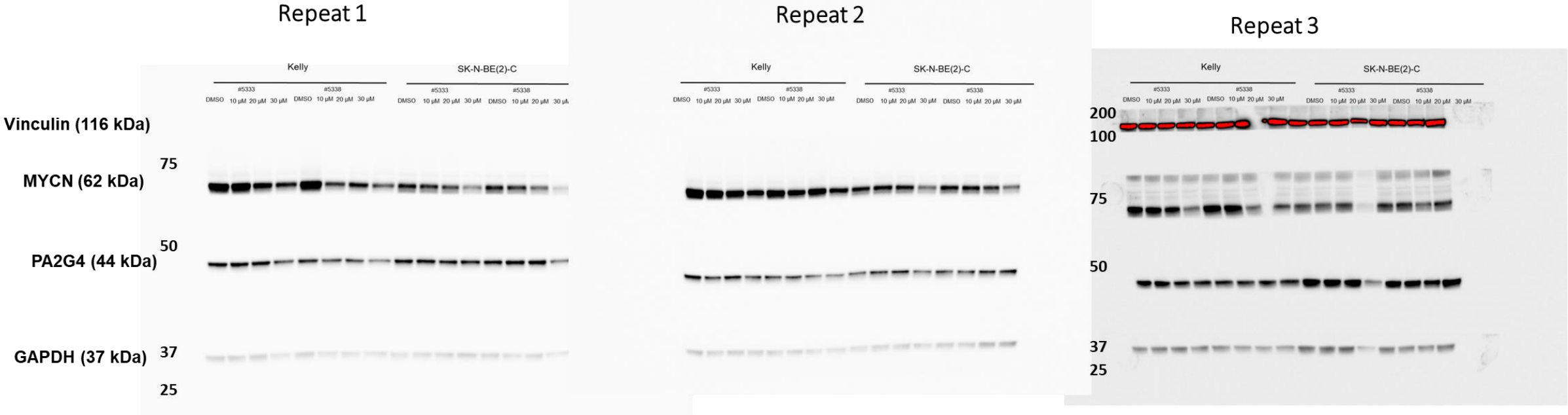

Figure 3 D & G

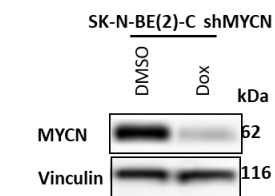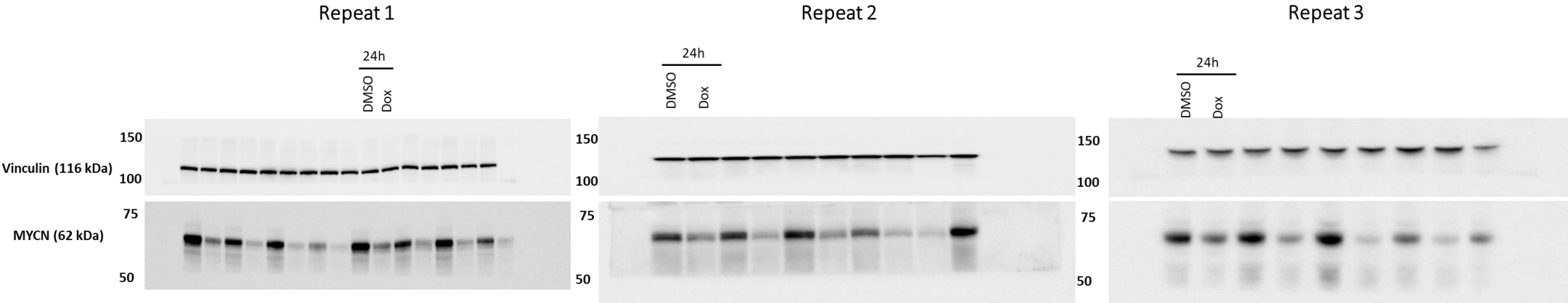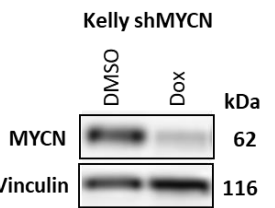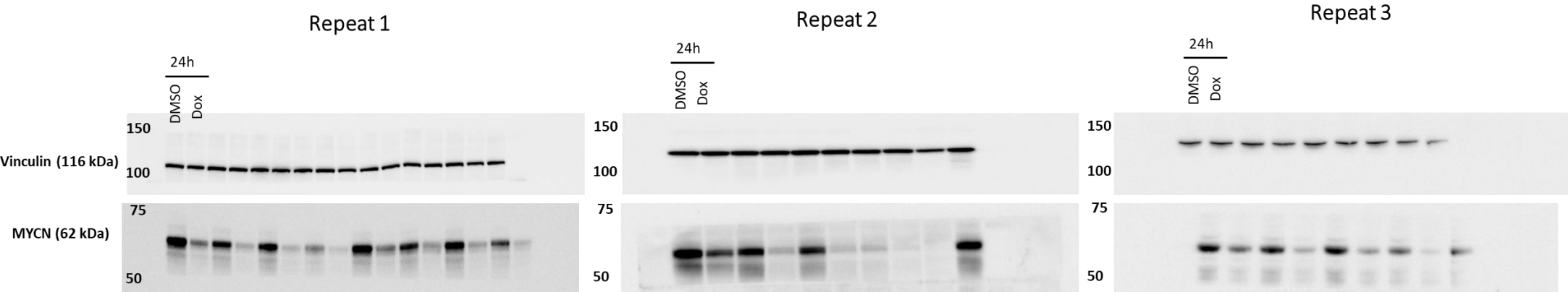

Figure 3H

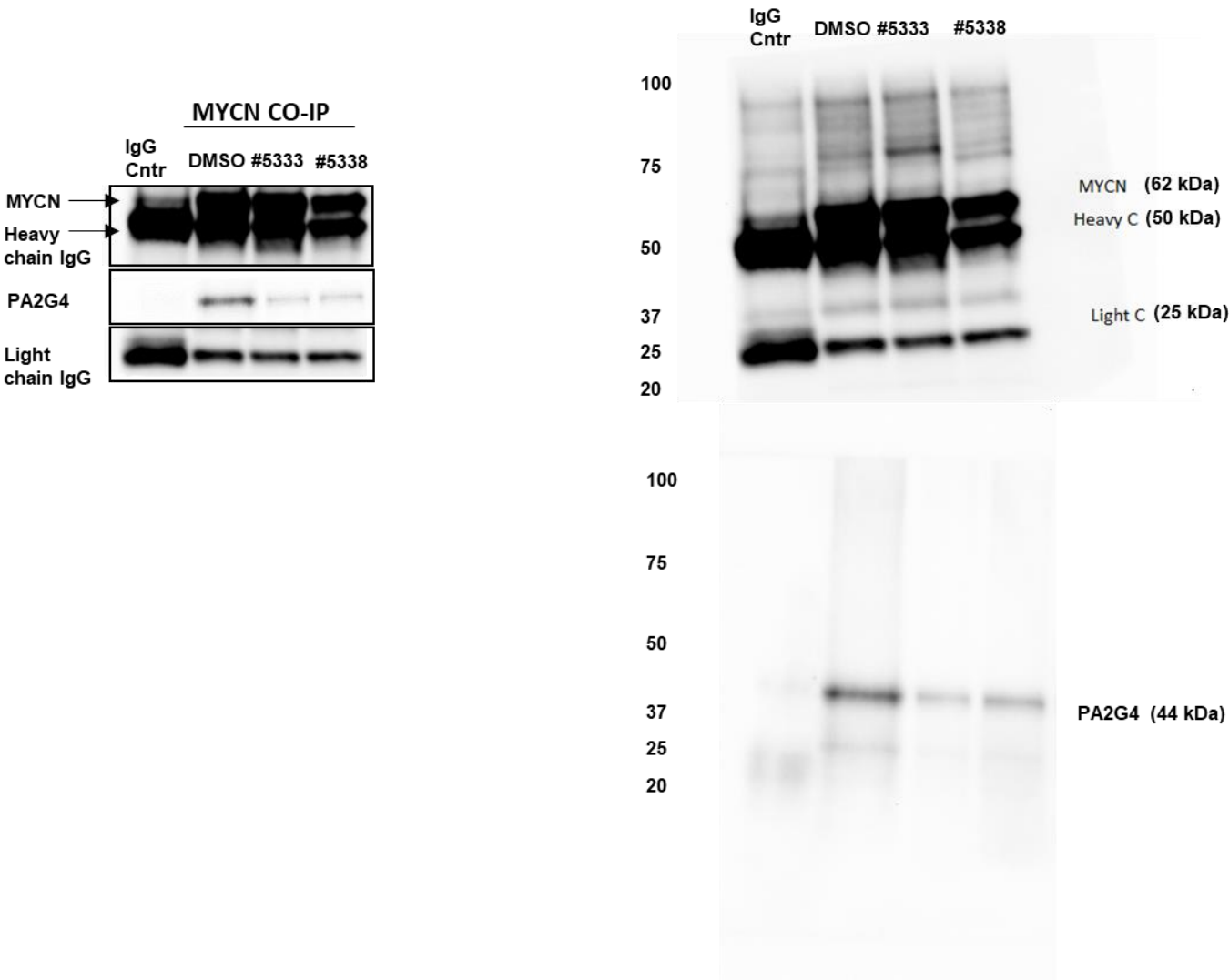

Figure 5A

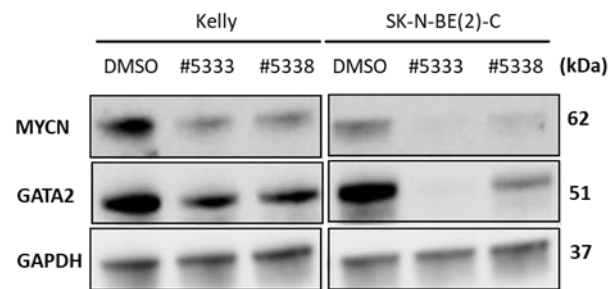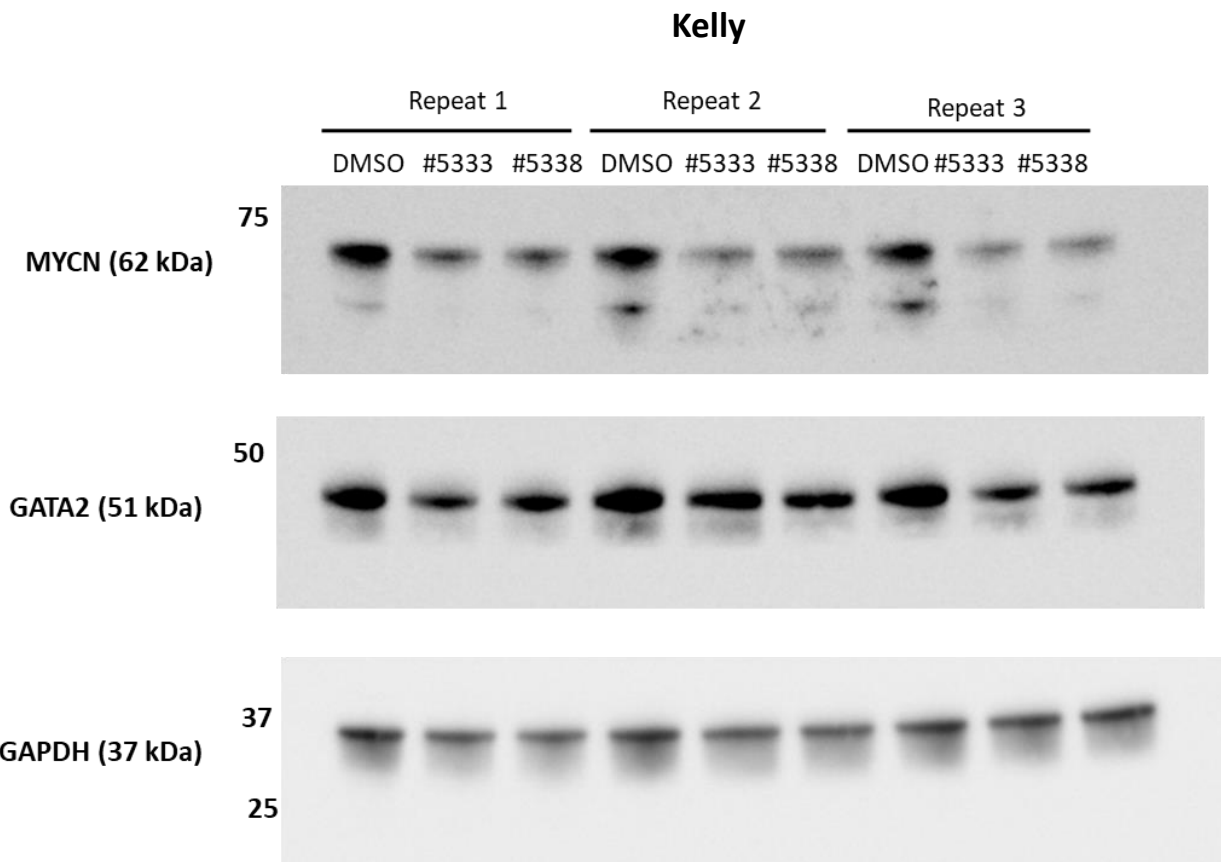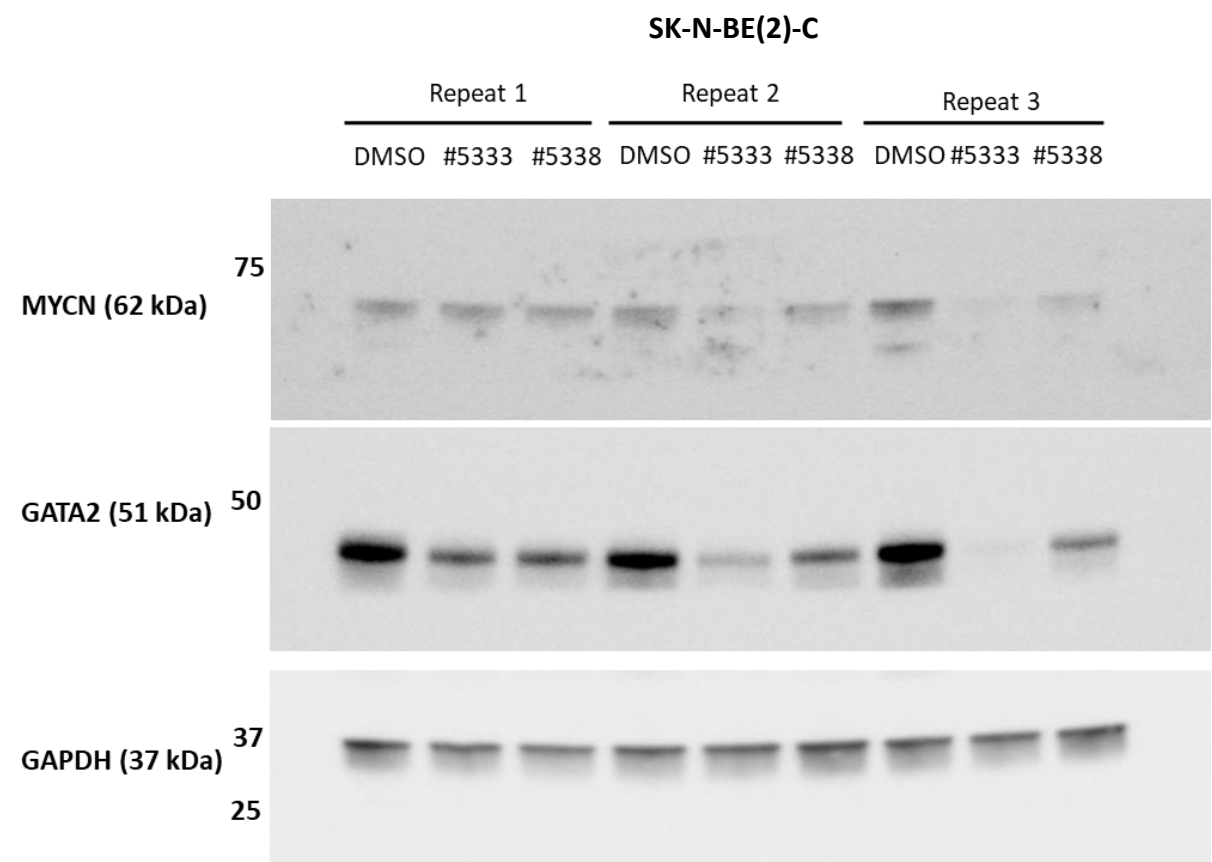

Figure 5C

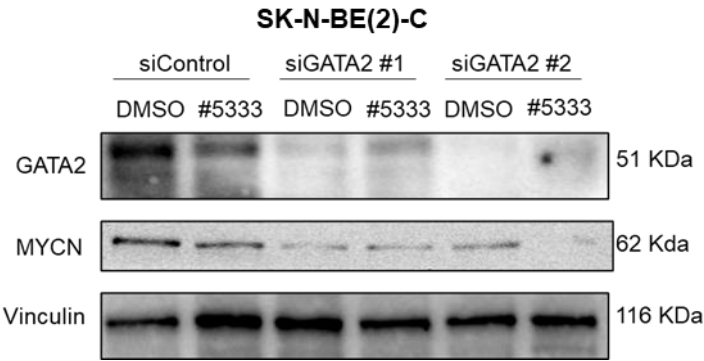

Repeat 1

Repeat 2

Repeat 3

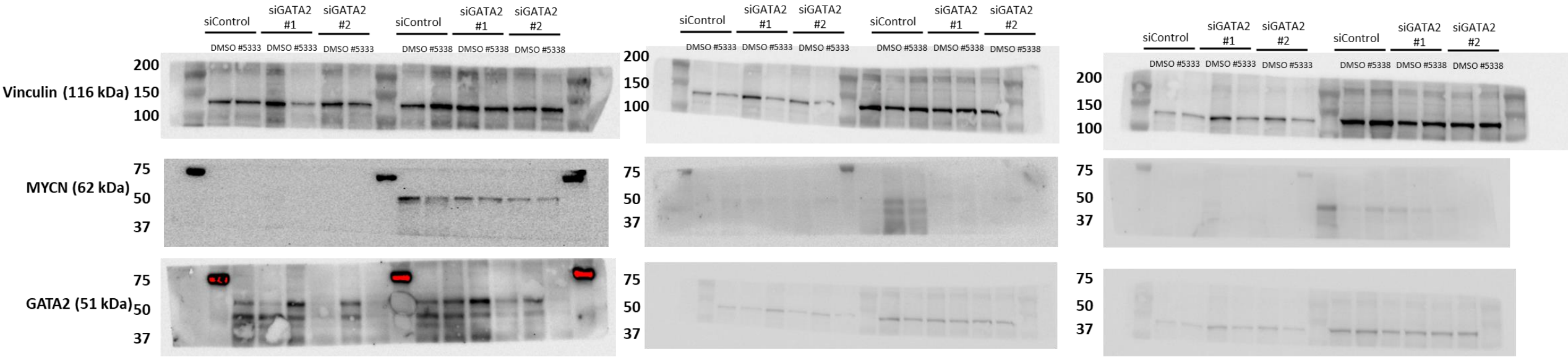

Supplement: Supplementary file 1 [file cancers-15-01822-s001.zip › cancers-2238268-original Western Blot figures.pdf]
